# Distribution of coronal plane alignment of the knee classification among Emirati patients with arthritic and healthy knees

**DOI:** 10.1002/jeo2.70535

**Published:** 2025-11-03

**Authors:** Ali Albelooshi, Muhieddine Hamie, Elhadi Babikir, Malak Almasri, Saeed Al Thani

**Affiliations:** ^1^ Orthocure Medical Center Dubai United Arab Emirates

**Keywords:** coronal alignment, coronal plane alignment of knee, kinematic alignment, knee arthritis, knee arthroplasty

## Abstract

**Purpose:**

Although the Coronal Plane Alignment of the Knee (CPAK) classification aids in characterising knee alignment phenotypes, it has drawbacks, such as misclassifying valgus knees as varus and allowing for significant aHKA variance within a single phenotypic. The proportions of each phenotype vary by area, despite the fact that worldwide CPAK distributions are largely stable. However, there is a lack of data from the Middle East, which restricts the system's use in planning total knee arthroplasty (TKA). Therefore, the objective of the present study was to ascertain the distribution of CPAK types among Emirati individuals with healthy knees and those with arthritis, as well as any variations in alignment traits between the two groups.

**Methods:**

A retrospective analysis was conducted on 795 Emirati patients (366 arthritic and 429 healthy knees) using standing long‐leg radiographs. Parameters measured included lateral distal femoral angle (LDFA), medial proximal tibial angle (MPTA), arithmetic hip‐knee‐ankle angle (aHKA), and joint line obliquity (JLO). Knees were classified into CPAK types based on these measurements.

**Results:**

The most common overall was CPAK II (neutral and apex distal) (40.4%), which was followed by CPAK I (varus and apex distal, 35.5%). In contrast to healthy knees, which were mostly CPAK II (45.5%), arthritic knees were primarily CPAK I (51.6%), suggesting a varus alignment tendency. MPTA was lower (84.75° ± 3.32 vs. 86.65° ± 2.51) and mean LDFA was greater (87.59° ± 1.86) in arthritic knees than in healthy knees (87.01° ± 1.96). With a mean aHKA of −2.85° ± 3.99, arthritis‐affected knees displayed more varus alignment than healthy knees, which had a mean aHKA of −0.37° ± 3.23.

**Conclusion:**

The prevalence of varus alignment in arthritic knees and the significance of region‐specific data are highlighted by this study, which offers the first CPAK distribution data for an Emirati population. These results can be used as a basis for future longitudinal and outcome‐based studies as well as to guide customised alignment tactics in TKA.

**Level of evidence:**

Level IV cross sectional descriptive study without control.

**Abbreviations**
aHKA angle: arithmetic hip‐knee‐ankle angle
CPAK: coronal plane alignment of the knee
JLO: joint line obliquity
KA: kinematic alignment
LDFA: lateral distal femoral angle
MA: mechanical alignment
MPTA: mechanical medial proximal tibial angle
TKA: total knee arthroplasty

## INTRODUCTION

A common orthopaedic surgery that offers substantial relief to patients with end‐stage knee osteoarthritis is total knee arthroplasty (TKA). Aiming for neutral limb alignment, mechanical alignment (MA) has historically been the accepted method [2, 3]. Recent developments, however, place an emphasis on a more customised approach, including kinematic alignment (KA), which aims to return the patient to their pre‐arthritic constitutional knee position. Although, KA is believed to more accurately mimic native joint kinematics and may enhance functional results and patient satisfaction, there is currently conflicting evidence and a lack of long‐term comparison data [6]. Because of this, the function of KA keeps changing as interest in customised TKA techniques increases.

TKA effectiveness depends on precise coronal limb alignment, which affects implant longevity and patient satisfaction. Developed by MacDessi et al., the Coronal Plane Alignment of the Knee (CPAK) classification offers a consistent framework by classifying knees into nine phenotypes according to two variables: arithmetic hip‐knee‐ankle angle (aHKA) and Joint Line Obliquity (JLO) [5, 6]. Despite being the norm for a long time, MA may ignore individual anatomical heterogeneity due to its one‐size‐fits‐all methodology. By taking into consideration the inherent variances in limb alignment, the CPAK classification provides a patient‐centred substitute for more individualised surgical planning. Clinically, knowledge of CPAK distributions can direct kinematic or mechanical alignment techniques that best restore natural knee function [1, 3].

Although CPAK makes preoperative alignment planning easier, the majority of research assessing the distribution of CPAK has been done on Western populations, with little information available from Middle Eastern cohorts. The use of CPAK in regional surgical planning is limited by this gap. Knowing population‐specific alignment patterns can help guide implant placement methods in TKA and guide the decision between kinematic and mechanical alignment procedures. This study aimed to ascertain the distribution of CPAK classification in Emirati individuals with knees in both healthy and arthritic. Creating this regional baseline will help with global alignment data and provide a basis for future studies, such as comparing clinical outcomes before and after total knee arthroplasty (TKA) based on changes in CPAK type.

## METHODS

This retrospective cohort study was conducted to review and analyse data for all Emirati patients who presented to Orthocure Medical Center during the period August 2023 to January 2025 with knee pain. A total of 795 participants were extracted from the center's electronic database. Inclusion criteria composed of the patients that had standing bilateral long leg radiographs while patients with incomplete data or did not have the required radiographs were excluded. Patients with lower limb bony deformity or previous fractures were excluded as well.

Seven hundred and ninety‐five knees of patients were divided into two cohorts, 366 arthritic knees (Ahlbäck grade 2–5) and 429 healthy (non‐arthritic) knees. Data were collected from electronic charts using data collection forms. The study was approved by the Institutional Review Board (IRB) of Dubai Health Authority (DSERC‐11/2024_11). The informed consent was obtained from all patients before data collection.

Bilateral long leg radiographs were performed by standardised method, maintaining the proper rotational position with bilateral patellae facing forward and similar shapes of both lesser trochanters and proximal tibiofibular joints. Radiological angles measurement was done by two fellows (MH and EA) using the hospital's PACS system. Angles measured were mLDFA, MPTA, aHKA and JLO.The mechanical femoral axis was defined as the line from the center of the femoral head to the center of the knee. The mechanical tibial axis was defined as the line from the center of the knee to the center of the ankle [12]. The mLDFA was defined as the lateral angle formed between the mechanical femoral axis and a line crossing the articular surface of the distal femur at its distal points on the lateral and medial sides [14], while the MPTA was defined as the angle formed by the tibial axis and a line drawn through the proximal articular surfaces of the medial and lateral tibial plateaus [10]. aHKA and JLO were obtained according to the algorithms described by MacDessi et al. [18]. Radiographic measurements were performed by two trained orthopaedic fellows using a standardised protocol and showed excellent intraclass correlation coefficient (ICC) value.

### Statistical analysis

The statistical analysis was performed by using RStudio software version 4.4.3. The continuous variables (age, mLDFA, MPTA, aHKA and JLO) were reported in median and interquartile range due to non‐paramteric distribution of data. The frequency and percentages were used to report categorical variables. The mean values of mLDFA, MPTA, aHKA and JLO were compared between the arthritic and the healthy knee cohort by Mann–Whitney *U* test. The scatter plot was used to visualise the CPAK classification in study population. *p*‐Value of < 0.05 was considered statistically significant.

## RESULTS

A total of 449 patients were included with 795 knees. Overall, mean age (in years) for all patients was 59.9 ± 15.89 years with 164 (37%) males and 285 (63%) females. Among all knee, 297 (37%) had a varus alignment, neutral in 403 (51%) and valgus in 95 (12%). The JLO was neutral in 116 (15%) of the patients and apex distal in 679 (85%). A total of 348 (77.5%) patients were operated for bilateral knees. Among them, windswept deformity was noted in 10 (3%) patients (right valgus and left varus in nine patients, similarly right varus and left valgus in 1 patient) (Table 1).

**Table 1:** General demographics.

Characteristics	Arthritics (*n* = 366)	Healthy (*n* = 429)
Laterality		
Right	189 (51.6)	213 (49.6)
Left	177 (48.3)	216 (50.3)
Alignment		
Neutral	144 (39.3)	259 (60.4)
Varus	196 (53.6)	101 (23.5)
Valgus	26 (7.1)	69 (16.1)
JLO		
Neutral	30 (8.2)	86 (20)
Apex distal	336 (91.8)	343 (80)

The femoral alignment was near‐normal with a minor valgus inclination, according to the alignment data, which showed a median LDFA of 87°. The proximal tibia appeared to have a slight varus inclination, as shown by the median MPTA of 86°. An overall moderate varus alignment of the knee is reflected in the median aHKA of −1.51° ± 3.80. A reasonably horizontal joint line with considerable population‐wide variability was indicated by the average Joint Line Obliquity (JLO), which was 173.96° ± 3.43.

With a median LDFA of 88° compared to 87° in healthy knees, arthritic knees had a larger distal femoral valgus angle. The arthritic group's MPTA (85°) was substantially lower than that of the healthy patients (87°), indicating a greater tibial varus. JLO was also slightly lower in arthritic knees (173° vs. 174°), and the median aHKA was more varus in arthritic knees (−3°) than in healthy knees (−0.0°). Table 2 lists the precise measurements.

**Table 2:** Measurements of healthy and arthritic knees.

Measurements	Arthritics (*n* = 366)	Healthy (*n* = 429)	*p*‐Value
LDFA (°)	88 (3)	87 (2)	<0.001
MPTA (°)	85 (4)	87 (4)	<0.001
aHKA (°)	−3 (4)	−0.00 (4)	<0.001
JLO (°)	173 (4)	174 (4)	<0.001

Abbreviations: aHKA, arithmetic hip‐knee‐ankle angle; JLO, joint line obliquity; LDFA, lateral distal femoral angle; MPTA, mechanical medial proximal tibial angle.

**Figure 1:**
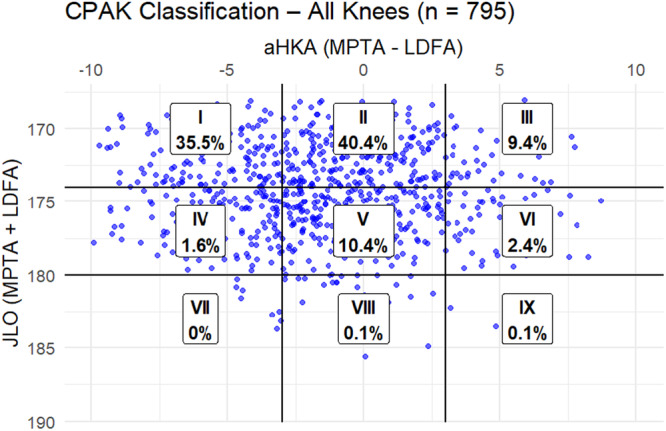
CPAK classification for all knees in study population. aHKA, arithmetic hip–knee–ankle angle; CPAK, coronal plane alignment of the knee; JLO, joint line obliquity; LDFA, lateral distal femoral angle; MPTA, medial proximal tibial angle.

Among all knees studied, CPAK II (neutral and apex distal) was most frequent (40.4%), followed by CPAK I (varus and apex distal) at 35.5%. CPAK III (valgus and apex distal) accounted for 9.4%. Neutral apex types (CPAK IV–VI) were less common, while proximal apex types (CPAK VII–IX) were rare ( < 0.3%). This distribution reflects a population trend toward distal apex and neutral‐to‐varus alignment phenotypes (Figure 1).

**Figure 2:**
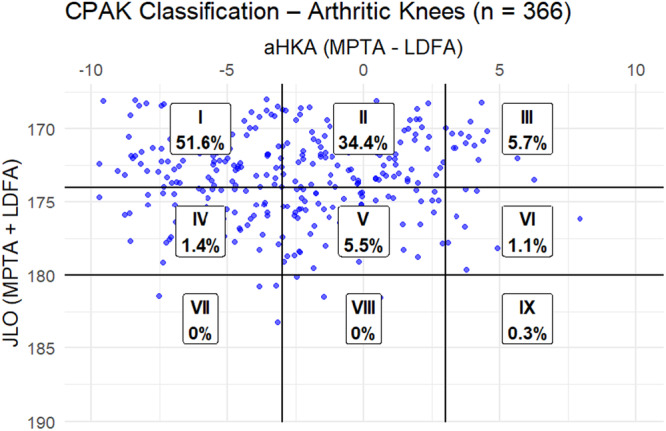
Distribution CPAK classification in the cohort of arthritic knees. aHKA, arithmetic hip–knee–ankle angle; CPAK, coronal plane alignment of the knee; JLO, joint line obliquity; LDFA, lateral distal femoral angle; MPTA, medial proximal tibial angle.

**Figure 3:**
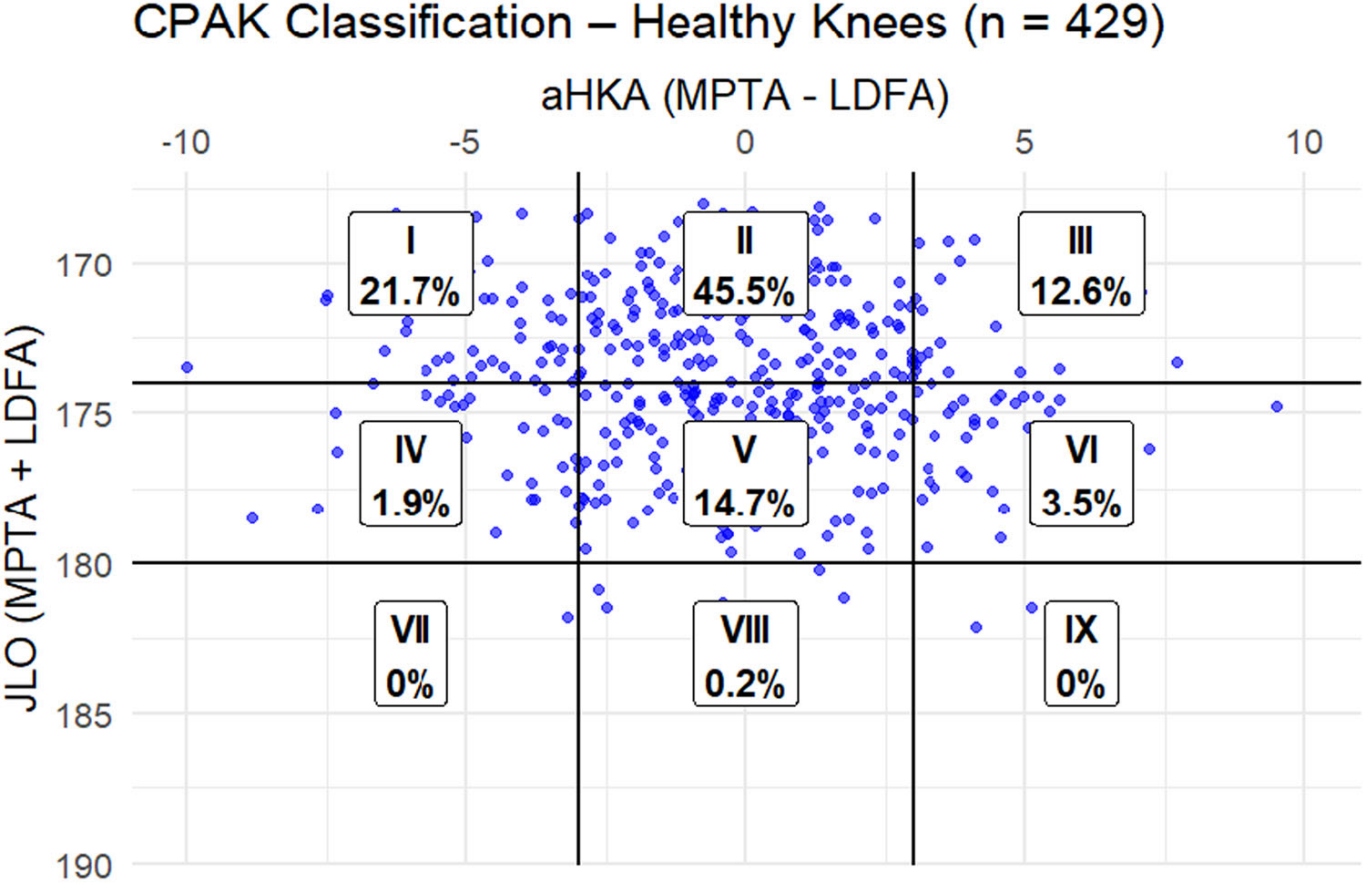
Distribution CPAK classification in the cohort of healthy knees. aHKA, arithmetic hip–knee–ankle angle; CPAK, coronal plane alignment of the knee; JLO, joint line obliquity; LDFA, lateral distal femoral angle; MPTA, medial proximal tibial angle.

In arthritic knees, CPAK I (varus and apex distal) was predominant at 51.6%, highlighting a strong varus alignment trend. CPAK II followed at 34.4%, and CPAK III was less frequent at 5.7%. Neutral apex types (CPAK IV–VI) appeared in less than 6% of cases, and proximal apex types were virtually absent (Figure 2). The most prevalent classification among healthy knees was CPAK II (45.5%), which was followed by CPAK I (21.7%) and CPAK V (14.7%). In 12.6% of instances, CPAK III (valgus and apex distal) was seen. There were fewer neutral and proximal apex types and no instances of CPAK VII or IX (Figure 3).

## DISCUSSION

The main findings of this study were that CPAK II (neutral and apex distal) was the most common overall (40.4%), followed by CPAK I (varus and apex distal, 35.5 in Emirati patients. Interestingly, CPAK I was more prevalent in arthritic knees (51.6%) whereas CPAK II was more prevalent in healthy knees (45.5%), indicating a distinct change toward varus alignment as arthritic development progressed.

CPAK classification is currently popularised worldwide as a simple system using long leg radiographs to identify knee phenotypes. Although it is comprehensive, it disregards axial and sagittal alignment which contributes to knee balance. There is paucity of studies that describe geographical variation in the prevalence of CPAK types among arthritic and healthy patients [13]. According to the current study, type II was most prevalent overall and among the healthy cohort, but type I was more prevalent among the arthritic group, which is likely due to the arthritis development. Additionally, there were very few individuals with apex proximal joint lines (type VII, VIII and IX). The CPAK distribution between healthy and arthritic knees was similar in the Australian cohort, according to MacDessi et al.'s findings (Type 5 and Type 2 were the most prevalent in both groups) [16]. In contrast, our Emirati population showed a shift toward greater varus phenotypes, specifically CPAK Types 1 and 4. With a larger physiological varus alignment reported in the Japanese population (mean varus 1.64° compared to 0.55° in Caucasians) [6], this pattern closely resembles that of the Japanese population. Because Emiratis are more likely to have varus phenotypes, our cohort's lower mean MPTA for arthritic knees when compared to Australian data may be explained. Similarly, Samant and Desai found that 56% of osteoarthritic knees in the Indian population had CPAK Types 1 and 4 (both varus), with a correspondingly lower MPTA of 85.7° as opposed to 88.3° in healthy knees [19]. The significance of population‐specific alignment data for planning total knee arthroplasty is highlighted by these geographical variances.

The CPAK distribution shown in the present Emirati population exhibits both noticeable parallels and variations when compared to data from other countries. CPAK II (neutral and apex distal) was the most prevalent type in a Belgian cohort, accounting for 39.2% of cases, which is comparable to our outcomes of 40.4%. In contrast, CPAK V (neutral and apex neutral) was less prevalent among Emiratis, occurring in just 10.4% of the whole population, whereas 15.4% of Belgians were affected [5]. The necessity for population‐specific alignment data to guide TKA design and results is further highlighted by the possibility that these discrepancies are due to genetic, lifestyle, or regional variances in native knee alignment [15]. According to Kobayashi et al., Japanese patients with arthritic knees are frequently categorised as type I, and when kinematic alignment is used, lower limb alignment may remain varus. The CPAK classification shows which patients are most likely to benefit from kinematic alignment or mechanical alignment techniques. The degree to which varus alignment can be tolerated is up for debate, although it has been noted that significant post‐operative varus alignment lowers functional scores and increases the risk of aseptic loosening following initial TKA [7].

The study conducted by Gao et al. supports the results that changes in bone alignment parameters are linked to the development of osteoarthritis (OA) [2]. In contrast to healthy knees (MPTA: 86.65°, LDFA: 87.01°), arthritic knees in the present research showed considerably lower MPTA (84.75°) and higher LDFA (87.59°), suggesting a shift toward varus deformity as OA progresses. The finding that OA progression may disrupt important bone landmarks like LDFA and MPTA is reinforced by these disparities, which support the idea that degenerative alterations affect both femoral and tibial alignment. When determining the location of the apex of the knee joint line obliquity or knee joint line orientation angle (the angle formed by the line parallel to the ground and the line tangential to the tibial condyles), Şahbat et al. indicate that the JLO calculation in the CPAK classification may be deceptive because the agreement between them is less than 50% in their study [9].

The majority of Indian patients with arthritis also had Type I knee phenotype, which includes apex‐distal joint line orientation and inherent varus alignment (294 knees, 58.8%). This was comparable to the Japanese population's CPAK alignment dispersion [11]. A significant percentage of constitutional varus was also found in another research of the Korean populace [17]. According to Song et al.'s evaluation of Korean and Caucasian populations, the majority of Koreans possessed constitutional varus alignment, and the MA was not neutral in the Korean population [17]. Additionally, Asian groups exhibit a larger spread and greater varus in lower limb alignment [6, 8, 17]. Furthermore, Graichen et al. showed that, in the majority of patients, a limited tibia‐first, gap‐balanced patient‐specific alignment (PSA) method could restore bone morphology and phenotypes following total knee arthroplasty. While valgus correction was less successful (37%), phenotypic restoration was attained in over 70% of straight‐legged and up to 94% of varus knees. All groups showed a considerable improvement in joint line convergence angle and a significant preservation of postoperative joint line obliquity. Following surgery, the CPAK classification changed toward a more neutral alignment, suggesting that normalisation was effective, especially in straight and varus knees. Results with significant varus and valgus abnormalities, however, were less predictable [4].

This study has a number of limitations that need to be noted. First, the patients who presented with degenerative osteoarthritis and knee discomfort were selected only from two institutes. The results could thus not be entirely indicative of the larger Emirati or UAE population. Additionally, this study did not analyse variability within individual CPAK subgroups or consider rotational alignment components, particularly relevant for CPAK types III and VI as these factors cannot be assessed using standard long‐leg radiographs. Therefore, larger, more varied samples from other UAE locations should be used in future studies to improve generalisability. Multicenter research with several assessors may be able to overcome this restriction and produce more reliable results. Future prospective large‐scale studies should expand on the current findings, with standardised imaging methods and long follow‐up, ideally over a 10‐year period.

## CONCLUSIONS

Significant disparities in knee alignment patterns between those with arthritis and those without are revealed by this study, which is the first to disclose the distribution of CPAK classification among Emirati patients. As the illness progressed, there was a noticeable change toward varus alignment, with CPAK I being more prevalent in arthritic knees and CPAK II being more prevalent in healthy knees. These results emphasise how crucial it is to use population‐specific alignment data when making clinical decisions regarding TKA. Establishing a regional baseline supports more customised and anatomically respectful alignment procedures in TKA by opening the door to future studies depending on CPAK types.

## AUTHOR CONTRIBUTIONS

**Ali Alblooshi**: Conceptualisation; data curation; formal analysis; investigation; methodology; project administration; supervision validation; writing–original draft preparation; writing–review and editing. **Muhieddine Hamie**: Data curation; formal analysis; investigation; methodology; project administration; visualisation; resources. **Elhadi Babikir**: Supervision validation; writing–original draft preparation; writing–review and editing. **Malak Almasri**: Data curation; formal analysis; investigation. **Saeed Al Thani**: Methodology; project administration.

## CONFLICTS OF INTEREST STATEMENT

The authors declare no conflicts of interest.

## ETHICS STATEMENT

The study was approved by the Institutional Review Board (IRB) of Dubai Health Authority (DSERC‐11/2024_11). Informed consent was obtained from all individual participants included in the study.

## Data Availability

The data supporting the findings of this study are available from the corresponding author upon reasonable request.
